# Effect of a 6-Month Functional Food Intervention on the Microbiota of Stunted Children in East Nusa Tenggara, Indonesia—A Randomized Placebo-Controlled Parallel Trial

**DOI:** 10.3390/foods14132218

**Published:** 2025-06-24

**Authors:** Ingrid S. Surono, Koen Venema, Subijanto Martosudarmo, Pratiwi D. Kusumo

**Affiliations:** 1 Food Technology Department, Faculty of Engineering, Bina Nusantara University, Jakarta 11480, Indonesia; isurono@binus.edu; 2 Centre for Healthy Eating & Food Innovation, Maastricht University, Campus Venlo, 5928 SZ Venlo, The Netherlands; 3 Beneficial Microbes^®^ Consultancy, 6709 TN Wageningen, The Netherlands; 4 Wageningen Food & Biobased Research, Wageningen University & Research, 6708 PG Wageningen, The Netherlands; 5 Department of Child Health, Faculty of Medicine, Universitas Airlangga, Surabaya 60286, Indonesia; subijantoms@gmail.com; 6 Faculty of Medicine, Universitas Kristen Indonesia, Jakarta 13630, Indonesia; pratiwi_d_k@yahoo.com

**Keywords:** stunted, nutritional status, Indonesian children under 5 year, gut microbiota, SCFA, *Faecalibacterium*, butyrate

## Abstract

We have previously shown a difference between the gut microbiota composition of stunted and non-stunted children in East Nusa Tenggara, Indonesia. The current study aimed to perform an intervention with a probiotic, *Lactiplantibacillus plantarum* IS-10506, and its UHT-treated postbiotic compared to placebo in order to accomplish catch-up growth in the stunted children, possibly through modulation of the gut microbiota. Apart from the maltodextrin (placebo), probiotic, and postbiotic in chocolate milk, all groups also received a functional and nutritional biscuit and had access to newly constructed water wells as well as soap to improve hygiene. The results show that independent of treatment, the stunted children had a significantly higher increase in height and zlen (corrected for age) compared with their age- and gender-matched controls but a significantly lower increase in weight. Several potential pathogenic taxa declined in all groups, among which was *Escherichia*/*Shigella* (adjusted.*p* = 6.44 × 10^−15^), but so did some beneficial taxa, such as *Bifidobacterium* and *Akkermansia*. *Faecalibacterium*, which was already higher in the stunted children at baseline, increased independent of treatment. Changes in the relative abundance of several taxa of the microbiota correlated with the changes in anthropometric measures. In conclusion, although there was no difference between the interventions, understanding the dynamics and the role of the gut microbiota in this process might allow healthcare providers to develop targeted nutritional strategies aimed at optimizing health outcomes for children at risk of stunting, thereby addressing a critical global health issue.

## 1. Introduction

Stunting, characterized by impaired linear growth in children, remains a serious global health issue, particularly in low- and middle-income countries, including Indonesia. Defined by a height-for-age Z-score (zlen) below −2 standard deviations of the World Health Organization (WHO) growth standards [1], stunting affects approximately 150 million children under five years old globally, with a significant proportion residing in Southeast Asia and sub-Saharan Africa [2,3]. In Indonesia, where malnutrition continues to affect child health and development, stunting prevalence remains unacceptably high, despite modest declines over the past decade [4,5]. In Indonesia, stunting still affected 4.6 million children in 2019, and this figure is still above the rate of stunted children in the broader Southeast Asia region (24.7%) [6].

The etiology of stunting is multifactorial, involving a complex interplay of nutritional deficiencies, recurrent infections, and suboptimal sanitation. Emerging evidence underscores the critical role of gut microbiota in stunting pathogenesis. The gut microbiota, a dynamic ecosystem of microorganisms inhabiting the gastrointestinal tract, contributes to nutrient metabolism, immune regulation, and gut barrier integrity. Dysbiosis, or imbalance in the gut microbial composition, has been implicated in reduced nutrient absorption, chronic inflammation, and impaired linear growth [7,8].

Stunted children frequently exhibit lower microbial diversity, an overabundance of pathogenic taxa such as *Escherichia*/*Shigella*, and diminished levels of beneficial butyrate-producing bacteria, including *Faecalibacterium* and *Ruminococcus* [3,9], although these taxonomic differences are not consistent in all studies. e.g., in our own baseline study, *Faecalibacterium* was increased in the stunted children [10]. These microbial alterations are often associated with environmental enteric dysfunction, a subclinical condition characterized by chronic intestinal inflammation and impaired barrier function, which further exacerbates growth faltering [2,11].

To address the problem of stunting, a comprehensive approach targeting both nutritional and environmental factors is necessary. Recent studies have highlighted the potential of interventions aimed at improving the gut microbiota composition as a strategy to promote healthy growth in stunted children [12,13,14,15,16]. Probiotics and postbiotics, which involve live microorganisms or inactivated microbial cells, respectively, have attracted attention as potential therapeutic strategies for restoring gut homeostasis in stunted children [11,17]. Certain probiotic strains, such as *Lactiplantibacillus plantarum* IS-10506, have been demonstrated to be safe and have immunomodulatory effects and the ability to enhance gut barrier integrity and nutrient absorption [18,19]. Similarly, postbiotics offer an alternative approach, delivering beneficial microbial fragments or metabolites directly without requiring viable microorganisms, thereby addressing limitations associated with probiotic survival in hostile gut environments [7].

East Nusa Tenggara is the province in Indonesia with the highest prevalence of stunting [20]. In our East Nusa Tenggara cohort of children under 5 years of age, we established a difference in microbiota in stunted versus non-stunted children at baseline [10]. Despite the common belief that stunting is largely irreversible after the child’s second birthday, we tested whether catch-up growth and correcting dysbiosis were possible. Therefore, a placebo-controlled, parallel 6-month intervention was carried out with UHT chocolate milk containing the microencapsulated probiotic *L. plantarum* IS-10506 or its UHT-treated postbiotic on top of hygienic measures. UHT chocolate milk with maltodextrin served as the placebo. All groups were also provided with a functional and nutritional biscuit [21].

## 2. Materials and Methods

### 2.1. Ethics Statement

Ethics were in accordance with the ethical standards of the responsible committee on human experimentation (institutional or regional) and with the Helsinki Declaration of 1975, as revised in 2000. The study protocol was approved by the Ethics Committee of the Research Institute of YARSI University under number 308/KEP-UY/BIA/XII/2022 and registered at ClinicalTrials.gov with identifier number NCT05119218. Parents or children’s caregivers received oral and written information and signed a letter of consent before children were included in the study.

### 2.2. Study Design

A parallel-arm, placebo-controlled study was conducted on children aged 3–5 years old with stunting (*n* = 100) and normal nutritional status (*n* = 100), at two locations: Kupang and North Kodi, in the East Nusa Tenggara (ENT) Province. Age (in months) and anthropometric measurements (height (or length) and weight) were recorded based on Department of Health Ministry of Indonesia regulations, and from these, BMI was calculated. In addition, the nutritional status of each child was quantified using the nutritional Z-scores recommended by the WHO, namely (length or) height for age (zlen); weight for age (zwei); and weight for length (zwfl). In addition, the Z-score for BMI for age (zbmi) was calculated. For stunting, the thresholds for zlen are ‘severely stunted’ (<−3 SD); ‘stunted’ (−3 SD to <−2 SD); ‘non-stunted’ (−2 SD to +3 SD); and ‘tall’ (>+3 SD) [1].

The children were randomized (age- and sex-matched) into 3 groups, receiving either placebo, probiotic, or postbiotic for a period of 6 months. To not potentially negatively affect the stunted children in the placebo group, they were all given a functional biscuit (biscuit F1 [21] formulated with 3% Moringa flour, 15% tempe flour, and 20% banana flour; total energy 99 kcal, fat 4 g, protein 2.1 g, carb 13.8 g, dietary fiber 10.4 g, sugar 3.4 g; manufactured by PT. Garudafood Putra Putri Jaya Tbk, Jakarta, Indonesia) and 125 mL of UHT chocolate milk (PT Ultrajaya Milk Industry and Company, Bandung, Indonesia). The placebo consisted of UHT milk with Avicel, encapsulated in alginate and CaCl_2_. The UHT postbiotic chocolate milk contained killed cells of the probiotic *Lactiplantibacillus* (L.) *plantarum* IS-10506, corresponding to 1 billion colony-forming units (CFUs) per pack before these cells were killed by standard UHT treatment. The same probiotic strain, but then alive, was encapsulated in alginate beads, as described before [22] and added at 1 billion CFUs in an aluminum sachet, which the parents or caretakers had to mix with the chocolate milk. This choice was made to prevent settling of the encapsulated cells in the milk carton during storage. The probiotic strain was encapsulated to increase its survival in the gastrointestinal tract [22]. Lunch and dinner were provided in a controlled diet to the children. On top of the nutritional intervention, the following hygienic measures were taken for all children: access to clean water was provided (by drilling water wells and installing pumps for drinking water), along with soap and training in hand washing.

### 2.3. DNA Isolation and Sequencing of the V3–V4 Region of the 16S rRNA Gene

Fecal samples were collected on site from stunted children and non-stunted children and immediately placed in a cooler with ice packs and shipped the same day to the lab in Jakarta on dry ice. In the lab, 0.5 g of the feces was mixed with 4.5 mL of Zymo buffer (Baseclear, Leiden, The Netherlands) and kept at room temperature prior to extraction of DNA. DNA extraction of feces samples was performed using the Quick-DNA™ Fecal/Soil Microbe Miniprep Kit (Zymo Research, Irvine, CA, USA) according to manufacturer’s instructions, using the Precellys 24 tissue homogenizer (Bertin Instruments, Montigny-le-Bretonneux, France), applying 3 cycles of 30 s each with 5 min cooling on ice in between. DNA was amplified with barcoding, pooled, and subsequently sequenced with an Illumina MiSeq sequencing system according to the manufacturer’s instructions (Illumina, San Diego, CA, USA), as described before [10]. Sequences were converted into FASTQ files using the BCL2FASTQ pipeline; primer sequences were removed, and quality trimming was applied based on the Phred quality score. QIIME 2 software (version 2022.2) was used for microbial analyses [23]. The sequences were classified using SILVA (version 132) as a reference 16S rRNA gene database. The amplified sequence variants (ASVs) table was filtered for those taxa that occurred in more than 20% of the total number of fecal samples. Blanks and controls were taken and behaved as expected.

### 2.4. Statistical Methods

Differences between groups in anthropometric measurements and Z-scores were investigated using Student’s *t*-test (between two groups) and an ANOVA (between all groups) followed by Tukey’s post hoc analysis. Non-parametric Spearman’s rank-order correlations were obtained between continuous variables (e.g., anthropometric data and microbiota composition). The Benjamini–Hochberg false discovery rate (FDR) was applied after the Spearman correlation test to correct for multiple comparisons. The non-parametric Kruskal–Wallis test was applied to categorical variables (e.g., being stunted or not and different interventions) and also corrected with the Benjamini–Hochberg FDR. Linear mixed modelling and random forest analysis were performed on the dataset as well. All of these calculations were performed using the software package R (4.3.1) (R Core Team, www.R-project.org/, accessed on 11 January 2024) in RStudio (version 2023.06.1, built 524), with the tidyverse package [24]. Boxplots were created using the package ggplot2 (part of tidyverse). Paired boxplots were created using the package ggpubr [25]. Heatmaps of correlations were created and visualized using the corrplot package [26]. Linear mixed modelling was performed with the packages lmer4 [27], lmer Test [28], and broom. mixed [29]. For random forest analysis, the random forest package was used [30], with parameters ntry = 25, ntree = 999, and 70% of the data in the training set. The variable importance was plotted using ggplot2 and ggpmisc [31]. Q-values (adjusted *p*-values after FDR) were considered significantly different at *q* < 0.1.

## 3. Results

### 3.1. Anthropometric Measures

**Figure 1:**
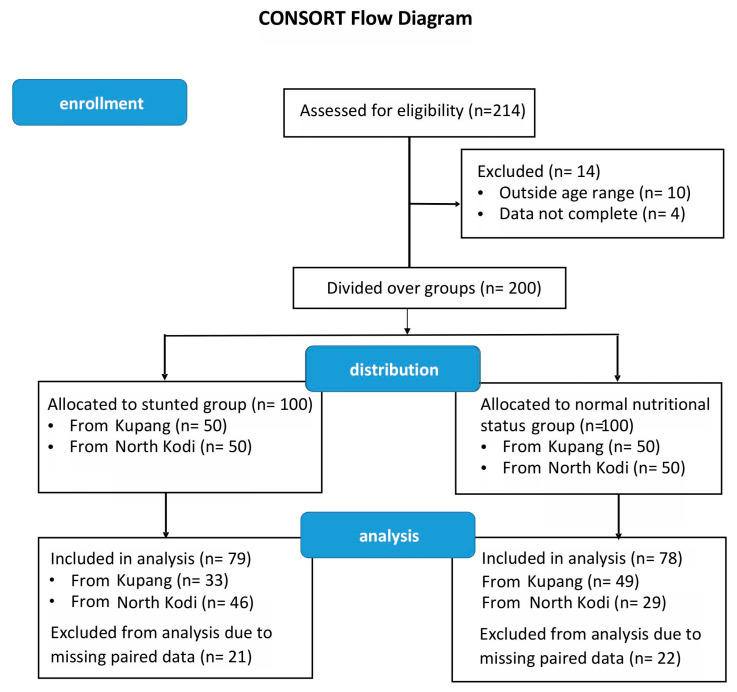
CONSORT flowchart of the study.

Figure 1 shows the CONSORT diagram of the study (Figure 1). For both stunted and non-stunted children, an initial total of 100 subjects were included. Baseline characteristics of these children were described before [10]. Due to missing paired sequence data, eventually, 79 stunted children and 78 non-stunted children were included in the analysis (Figure 1). These were essentially age- and gender-matched, although after removing subjects with paired missing data, age showed a significantly higher value for the stunted group. Table 1 shows the anthropometric data of those children at baseline and after the 6-month intervention, for which a complete dataset was collected at both time points. At baseline, the average Z-score for length (zlen) for the stunted children was −2.67 ± 0.51, ranging from −2.00 to −4.25. As observed before in our Java cohort [32], the non-stunted children were on average also shorter than the WHO reference standard, with an average of −1.12 ± 0.80, and a range of −1.99 to 1.15. Thus, despite being considered to have normal length according to the WHO, only 9 of 78 children were at or above the WHO reference zlen of 0.

The intervention consisted of increased access to clean water (essentially ensured by newly constructed drilled water wells) and providing functional biscuits [20] and chocolate milk for all children. To the chocolate milk either maltodextrin (placebo), the UHT-killed probiotic, or the probiotic *L. plantarum* IS-10506 was added. Therefore, the effects in the placebo group could be due to the hygienic measures in combination with the provided functional biscuit and chocolate milk.

**Figure 2:**
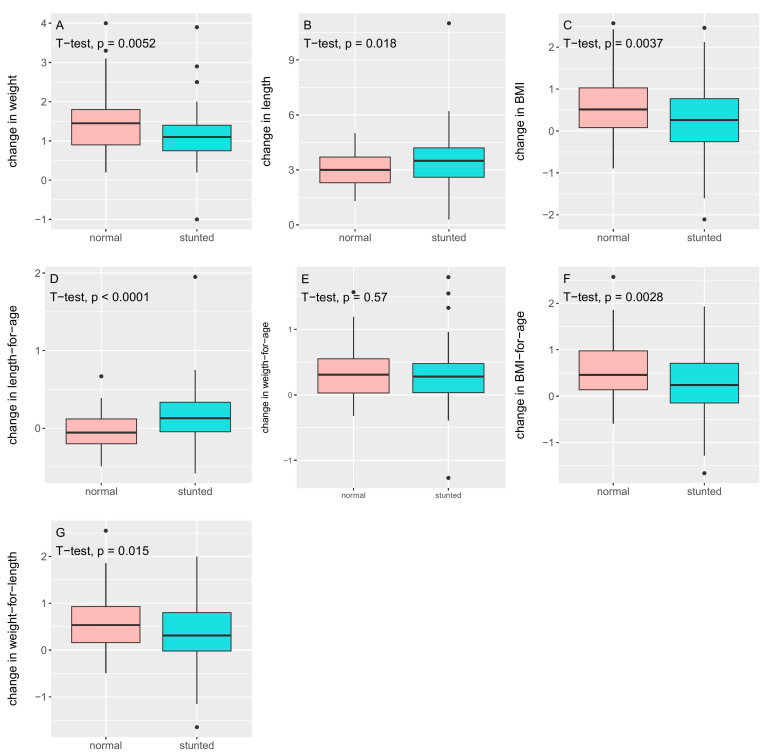
Change (delta) in anthropometric parameters (**A**) weight, (**B**) length, (**C**) BMI, (**D**) zlen, (**E**) zwei, (**F**) zbmi, and (**G**) zwfl after the 6-month intervention for the non-stunted and stunted group.

Paired boxplots of the changes in the anthropometric parameters are provided in Supplemental Figure S1. As expected, all children grew in length and grew heavier (Table 2 and Figure 2 for change in anthropometric parameters). However, the stunted children remained shorter (length, zlen) and lighter (weight, zwei) than the non-stunted children after the 6-month intervention (Table 1). Despite this, the stunted children grew significantly more in length (3.55 ± 1.51 cm) than the non-stunted children (3.06 ± 0.93 cm, *p* = 0.018; Table 2 and Figure 2), although the difference was small. On the other hand, the non-stunted children gained significantly more weight (stunted 1.15 ± 0.66 kg versus non-stunted 1.46 ± 0.74 kg, *p* = 0.005; Table 2 and Figure 2), leading to a trend in significant differences for BMI (and zbmi) after the intervention (Table 1). Due to these changes in weight and length, the weight-for-length Z-score (zwfl) became significantly different between the two groups after the intervention (Table 1). Strikingly, the non-stunted children on average had a negative change in zlen (Table 2 and Figure 2), whereas the stunted children, in line with their bigger increase in length, had a positive change in zlen. The difference was highly significant (*p* < 0.0001).

The changes described above were independent of the interventions (placebo, probiotic or postbiotic). The ANOVA did not show any significant differences in the changes in anthropometric parameters between the three groups after the interventions (Supplemental Table S1a–g). Only when the postbiotic group was combined with the probiotic group (from which the postbiotic was derived) was the difference in weight after the intervention significantly higher than placebo (*p* = 0.05), with a trend in BMI (*p* = 0.07) and zbmi (*p* = 0.07). The two-factor ANOVA on the stunted and non-stunted groups split up by treatment showed significance for change in weight (*p* = 0.015), change in BMI (*p* = 0.0067), change in zlen (*p* = 0.0008), change in zbmi (*p* = 0.0055), and change in zwfl (*p* = 0.021) (Supplemental Table S2a–g). The Tukey post hoc analysis indicated that this was primarily due to differences within the postbiotic group, which showed higher weight gain (non-stunted: 1.59 ± 0.73, stunted: 1.04 ± 0.84; *q* = 0.05), higher BMI gain (non-stunted: 0.77 ± 0.69, stunted: 0.12 ± 0.80; *q* = 0.023), higher delta zlen (non-stunted: 0.71 ± 0.63, stunted: 0.14 ± 0.70; trend *q* = 0.051), higher delta zbmi (non-stunted: 0.74 ± 0.63, stunted: 0.18 ± 0.69; *q* = 0.015), and higher delta zwfl (non-stunted: 0.74 ± 0.61, stunted: 0.25 ± 0.70; *q* = 0.044) for the non-stunted children compared to the stunted children (Supplemental Table S2a–g).

### 3.2. Microbiota Composition

At baseline, only three taxa were significantly different between non-stunted and stunted children as we reported before [10]: *Lachnoclostridium*, *Faecalibacterium* and *Veillonella* (Supplemental Figure S2). Of the 92 taxa that were prevalent in at least 20% of the samples, based on a Kruskal–Wallis analysis with Benjamini–Hochberg FDR correction, 85 taxa were significantly different between baseline and after intervention when split up in stunted and non-stunted children groups (*q* < 0.1). The most significant (by *q*-value and arguably also by biological relevance) was *Escherichia/Shigella* (the sequence of the V3–V4 region cannot discriminate between these two genera). The significant difference (*q* = 6.44 × 10^−15^) was between baseline and after intervention (endline) for all groups combined.

**Figure 3:**
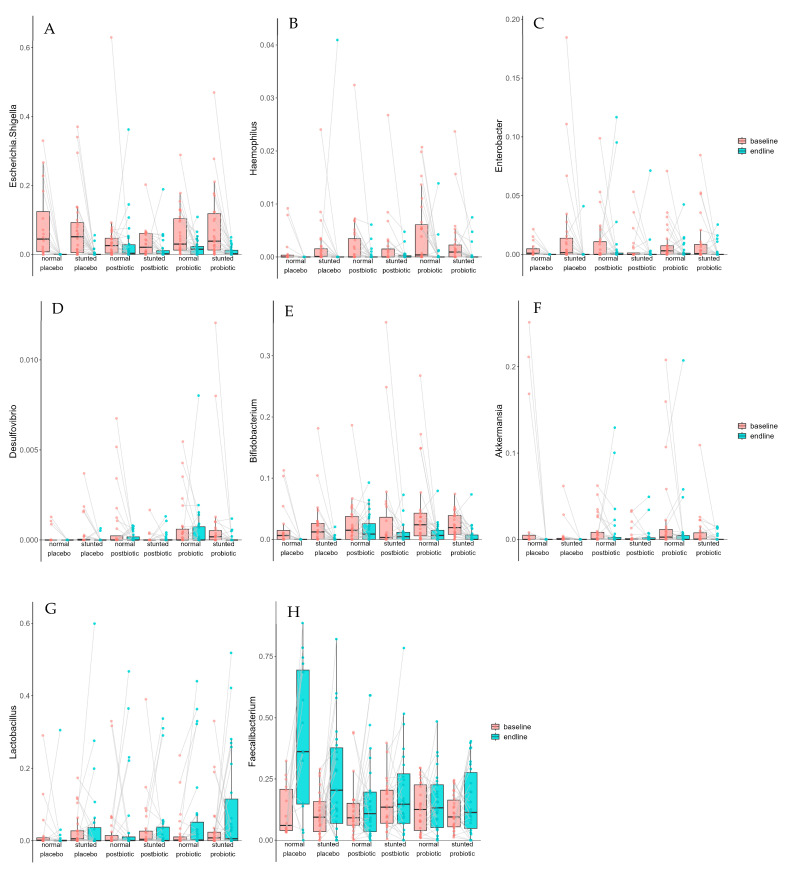
Response (in relative abundance) of several relevant taxa at baseline and at the end of the intervention (endline) with the three treatments, split up by children that were stunted or not: (**A**) *Escherichia/Shigella* *, (**B**) *Haemophilus* *, (**C**) *Enterobacter* *, (**D**) *Desulfovibrio*, (**E**) *Bifidobacterium* *, (**F**) *Akkermansia*, (**G**) *Lactobacillus*, and (**H**) *Faecalibacterium* *. The asterisk (*) indicates that these taxa were also significant when linear mixed modelling was applied (Table 3).

**Figure 4:**
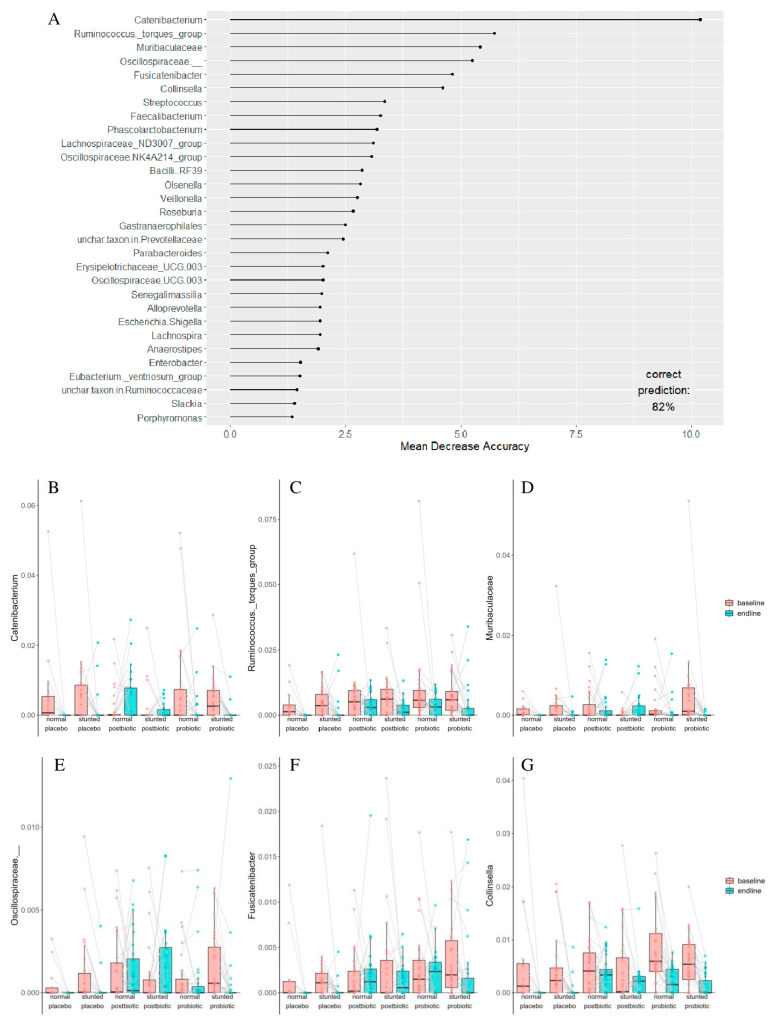
(**A**) Variable importance plot and response (in relative abundance) of taxa with a mean decrease accuracy of >4 in the random forest analysis at baseline and at the end of the intervention (endline) with the three treatments, split up by children that were stunted or not: (**B**) *Catenibacterium* *, (**C**) *Ruminococcus torques group* *, (**D**) *Muribaculaceae* *, (**E**) uncharacterized taxon in the family Oscillospiraceae, (**F**) *Fusicatenibacter* *, and (**G**) *Collinsella* *. The asterisk (*) indicates that these taxa were also significant when linear mixed modelling was applied (Table 3).

At baseline, *Escherichia/Shigella* was prevalent at an average of 6.4% relative abundance (RA) and not significantly different between stunted and non-stunted children (6.3% ± 9.7% for non-stunted children; 6.6% ± 9.1% for stunted children). After the 6-month intervention, the prevalence dropped to 1.5% RA, with the largest drop in the placebo group, but there were no differences in response between stunted and non-stunted children (2.0% ± 5.0% for non-stunted children; 1.1% ± 2.6% for stunted children; Figure 3A). On average, 28 taxa increased in relative abundance, whereas 64 decreased. Apart from *Escherichia/Shigella*, other potential/opportunistic pathogens decreased as well, such as *Haemophilus* (*q* = 2.0 × 10^−9^; Figure 3B), *Enterobacter* (*q* = 7.6 × 10^−7^; Figure 3C), and *Desulfovibrio* (*q* = 0.04; Figure 3D). Unfortunately, several taxa that are normally considered beneficial for health were reduced as well, such as *Bifidobacterium* (*q* = 9.7 × 10^−9^; Figure 3E), *Akkermansia* 7.7 × 10^−5^; Figure 3F), and several butyrate producers. On the other hand, *Lactobacillus* (*q* = 0.054; Figure 3G) and *Faecalibacterium* increased (*q* = 0.007), the latter primarily in the placebo group (Figure 3H). Linear mixed modelling also indicated some of these taxa to be significantly different. These are indicated with an * in the legend of Figure 3. Table 3 shows all taxa identified by linear mixed modelling to be significantly linked to baseline vs. after intervention (endline) or to be associated with the postbiotic or probiotic group. Paired boxplots (that are not already in Figure 3 and Figure 4) are in Supplemental Figure S3.

Random forest analysis was used to predict to which group a child belonged (stunted or non-stunted, one of the three interventions) based on the change in microbiota composition compared to baseline (Figure 4A). *Catenibacterium* was the taxon that explained most of the predictions into groups, primarily because it was high in the baseline placebo and probiotic groups (both stunted and non-stunted children) and was basically absent after intervention in these groups, whereas the reverse was true for the postbiotic groups (stunted and non-stunted), where *Catenibacterium* was basically absent at baseline but increased after the intervention (Figure 4B). Focusing on the taxa with a mean decrease accuracy of 4 or more, it can be observed that the *Ruminococcus torques* group decreased in all groups, but more so in the placebo treatment (Figure 4C). Moreover, *Muribaculaceae* was reduced in all groups after the intervention (Figure 4D). An uncharacterized taxon in the family *Oscillospiraceae* was also identified by random forest to be predictive for the groups. It was reduced in both placebo-treated groups and in both probiotic-treated groups, whereas it was slightly increased in both postbiotic-treated groups (Figure 4E). *Fusicatenibacter* was reduced in both placebo-treated groups and in the stunted probiotic-treated group, whereas it was increased in both postbiotic-treated groups and the non-stunted probiotic-treated group (Figure 4F). Lastly, *Collinsella* was reduced in all groups, except the stunted postbiotic-treated group (Figure 4G). Taxa with an asterisk in Figure 4 were also identified by linear mixed modelling to be significant (Table 3).

### 3.3. Correlation Between Anthropometric Measures and Changes in Microbiota Composition

**Figure 5:**
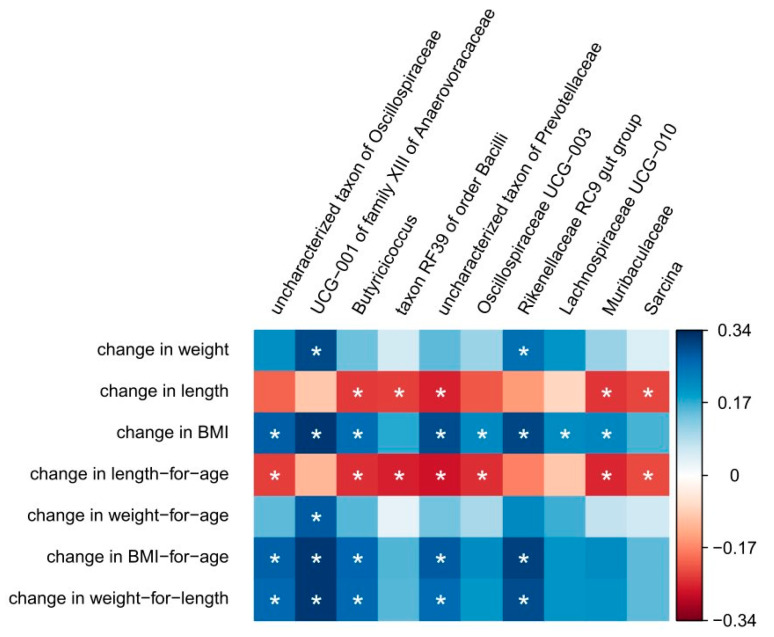
Heatmap of the significant Spearman correlations between taxa and anthropometric measures. * indicates significance (*q* < 0.1). A blue color indicates a positive correlation, red a negative correlation.

The change in anthropometric measures from baseline to after intervention (endline) was correlated with the change in microbiota composition. Figure 5 shows the taxa that have one or more significant correlations with the anthropometric measures. Two of the taxa identified by random forest, the uncharacterized taxa in the family Oscillospiraceae and Muribaculaceae, both showed a negative correlation with the change in zlen (*q*- and rho-values in Supplemental Table S3). Moreover, *Butyricicoccus*, taxon RF39 of order Bacilli, an uncharacterized taxon of Prevotellaceae, *Oscillospiraceae UCG−003,* and *Sarcina* also showed a negative correlation with the change in zlen (Figure 5).

## 4. Discussion

The intricate relationship between dietary changes and microbiota composition plays a vital role in influencing growth outcomes in stunted children. Recent studies highlight that modifying dietary intake can significantly alter gut microbiota, which in turn impacts nutritional absorption and immune, metabolic, and gut health [8,12,13,14,17]. Dysbiosis, or an imbalance in the gut microbial community, has been linked to undernutrition and impaired growth, suggesting that the gut microbiota may play a pivotal role in the pathophysiology of stunting [33]. Research indicates that alterations in gut microbiota composition can precede observable stunting, highlighting the potential for early interventions to mitigate growth impairment [34]. Moreover, specific microbial metabolites, particularly short-chain fatty acids (SCFAs), are crucial for maintaining gut health and regulating growth, further establishing the gut microbiota’s influence on child development [35,36]. Furthermore, hygienic practices significantly impact the composition and diversity of gut microbiota, which are crucial for the growth and development of children, particularly those at risk of stunting. Poor hygienic conditions can lead to the disruption of beneficial microbial communities, resulting in dysbiosis—a state that has been linked to malnutrition and stunted growth. Effective hygiene interventions, including improved sanitation and food safety, can foster a healthier microbiota, which subsequently supports optimal nutritional outcomes in children. Recent studies highlight the interplay between hygiene, nutrition, and gut microbiota, suggesting that enhancing children’s microbiota through hygienic measures may help mitigate growth impairments associated with chronic malnutrition [37,38,39].

At baseline, our cohort contained high numbers of *Escherichia*/*Shigella*, irrespective of nutritional status [10]. Moreover, the stunted children contained more Gram-negative taxa and more potential pathogens [10]. The drastic reduction of *Escherichia*/*Shigella* upon intervention in all groups is likely reflective of the hygienic measures that were taken in the study, although the role of the functional biscuit cannot be ruled out, as also the placebo-treated children showed this reduction. Together with a reduction in other potential pathogens, such as *Enterobacter*, *Haemophilus* and *Desulfovibrio*, it is tempting to speculate that the (gut) health of the children was improved. Future studies will have to shed light on this.

The initial baseline microbiota composition did not significantly predict response to the intervention. Numerous taxa changed. The reduction in several of the Gram-negative taxa could have beneficial effects on immunological and metabolic functions. However, taxa that are also considered beneficial, such as *Bifidobacterium* and *Akkermansia,* were reduced. Several probiotic strains of *Bifidobacterium* species have been shown to be immunomodulatory e.g., [40,41]. As sequencing analysis of the V3–V4 region of the 16S rRNA gene only allows identification at the genus level, it is currently unclear which species of *Bifidobacterium* were reduced and what the effect of that is on immunological and metabolic functions. While *Akkermansia* is negatively correlated with overweight [42], its role in stunting has not been investigated before. *Faecalibacterium*, considered a biomarker for a healthy gut microbiota [43], was increased in all treatments. It has been shown to have anti-inflammatory effects (among others) due to production of butyrate and an extracellular MAM-protein [43,44].

Several studies have shown that probiotics have a limited effect on the composition of the gut microbiota [45,46,47]. Some studies have suggested that probiotics may impact the function of colonizing microbes, although this needs to be further studied. An alternative hypothesis is that probiotics may promote homeostasis of the gut microbiota, rather than change its composition [46]. In our study there were limited changes in the gut microbiota of the pro- and postbiotic group compared to the placebo. Even *Lactobacillus* levels did not rise significantly in the probiotic-treated children, despite protective microencapsulation [22]. Both groups had an equal distribution of children showing either an increase or a decrease (non-stunted and stunted; Figure 3G), but this was similar in the postbiotic and placebo groups. As the postbiotic was the UHT-killed probiotic, no effect on *Lactobacillus* counts was expected. Nevertheless, both treatments may affect host health by affecting, e.g., immune or metabolic parameters in the upper GI tract, as has been shown for this probiotic in numerous previous studies [18,19,48,49,50,51,52]. This was not investigated in the current study. Here, we aimed to study the effect of the interventions on growth. Only the postbiotic group showed measurable improvement (Supplementary Table S2), where the change in zlen for the stunted children was significantly higher than that of the non-stunted children. The probiotic treatment did not show significant differences. It is unclear why the postbiotic showed slightly more favorable trends. Beyond reducing the pathogen load in the children, as observed for *Escherichica*/*Shigella*, the probiotic/postbiotic could beneficially affect the immune system. The immune system, when activated (usually due to infections), consumes a lot of energy that cannot be used for linear growth. This might have been a mechanism by which this particular probiotic (and/or its postbiotic) could have contributed to reduced lag in growth. Probiotics should be alive upon ingestion. In tropical countries like Indonesia, distribution of cooled products containing live probiotics to remote areas such as East Nusa Tenggara is challenging. The use of inactivated cells of the probiotic can therefore be of paramount importance for this kind of application. Several studies have shown that inactivated cells (postbiotics) can be as efficacious as the live probiotics themselves e.g., [53]. Unfortunately, the current study could not discriminate the effect of the intervention with both ‘-biotics’ above the simultaneous intervention with the functional biscuit and hygienic measures.

Overall, when all treatments were combined, the stunted children grew a few centimeters more in those 6 months than the non-stunted children, which led to a highly significant higher increase in zlen (*p* < 0.0001). Although the change was minimal and surely did not lead to the stunted children catching up with the non-stunted children (the difference in height was, on average, 5.1 cm at baseline vs. 4.61 cm after the intervention), still they showed some catch-up due to the treatments. This is likely more due to the hygienic measures in combination with the functional biscuit and the provided chocolate milk (in which the placebo was also provided), then the pro- or postbiotic treatment. It is tempting to speculate that this +0.5 cm in 6 months may translate to meaningful better health later in life, certainly if this catch-up growth can be maintained, but this remains to be seen. It is unknown whether changes in the microbiota and anthropometric measures were sustained after cessation of the intervention, except that the little bit of catch-up growth that was seen for the stunted children will likely remain. The rate of catch-up may, however, have dropped again. This should be investigated in follow-up studies. There were no taxa that showed significant positive correlations with a change in zlen and/or height, but several taxa showed negative correlations with a change in these measures. These might be taxa that can be targeted by nutritional interventions early in life to mitigate the retardation in growth in stunted children. Which interventions are successful in targeting these taxa remain to be seen.

A strength of the study was that food intake was controlled, in the sense that all lunches and dinners were provided. Nevertheless, we could not isolate the effects of the probiotic/postbiotic interventions from other simultaneous interventions such as hygienic interventions (e.g., clean water, soap, and handwashing education), which could have been confounding factors. The placebo group also showed improvements, suggesting that non-specific intervention components (e.g., improved nutrition and hygiene) likely contributed substantially to outcomes. Including a true control group without nutritional or hygiene enhancements; using factorial or crossover designs to better understand and unravel each component’s contribution; and measuring health biomarkers (e.g., SCFAs, inflammatory markers) to differentiate functional effects more clearly in future trials are necessary steps. The influence of sex or using finer age intervals (e.g., 3 instead of 5 years) on growth or microbiota changes was not explored. These factors can explain heterogeneity in outcomes and modulate response to the intervention. However, this study used a similar setup as our earlier study in Java, with the same age interval. During that study, we did not observe any indications that a smaller age interval would have reduced heterogeneity.

The cyclical nature of stunting, where stunted mothers are likely to give birth to stunted children, underscores the intergenerational transmission of this condition [54] and the importance of tackling this issue. Ultimately, understanding the dynamics and the role of the gut microbiota in this process allows healthcare providers to develop targeted nutritional strategies aimed at optimizing health outcomes for children at risk of stunting, thereby addressing a critical global health issue. Improving the gut microbiota in conjunction with re-nutrition techniques has the potential to ameliorate growth and development impairments in these stunted children. Our future studies will focus on this.

## Figures and Tables

**Table 1:** Anthropometric scores of the children at baseline and after the 6-month intervention.

	Baseline			After Intervention		
	Stunted	Non-Stunted	*p*-Value	Stunted	Non-Stunted	*p*-Value
	*n* = 79	*n* = 78		*n* = 79	*n* = 78	
M/F (%)	47/53	46/54	1 ^†^	47/53	46/54	1 ^†^
age (months)	46.76 ± 4.82	44.43 ± 5.07	**0.004**	52.76 ± 10.82	50.43 ± 11.07	**0.004**
weight (kg)	11.79 ± 1.09	13.13 ± 1.50	**<0.0001**	12.93 ± 1.23	14.60 ± 1.85	**<0.0001**
length (cm)	91.01 ± 3.20	96.11 ± 4.05	**<0.0001**	94.56 ± 3.53	99.17 ± 3.98	**<0.0001**
BMI (kg/cm^2^)	14.22 ± 1.00	14.20 ± 1.17	0.91	14.46 ± 1.05	14.80 ± 1.16	0.056
zlen	−2.67 ± 0.51	−1.12 ± 0.80	**<0.0001**	−2.18 ± 0.60	−0.80 ± 0.81	**<0.0001**
zwei	−2.39 ± 0.63	−1.36 ± 0.84	**<0.0001**	−1.89 ± 0.66	−0.79 ± 0.89	**<0.0001**
zbmi	−0.90 ± 0.81	−0.98 ± 0.98	0.63	−0.68 ± 0.83	−0.44 ± 0.89	0.087
zwfl	−1.20 ± 0.80	−1.05 ± 0.98	0.3	−0.87 ± 0.83	−0.47 ± 0.90	**0.005**

^†^ Pearson’s Chi-squared test with Yates’ continuity correction; other analyses: *t*-test.

**Table 2:** Changes in anthropometric scores of the children after the 6-month intervention.

	Stunted	Non-Stunted	*p*-Value
	*n* = 79	*n* = 78	
Δ weight (kg)	1.15 ± 0.66	1.46 ± 0.74	**0.005**
Δ length (cm)	3.55 ± 1.51	3.06 ± 0.93	**0.018**
Δ BMI (kg/cm^2^)	0.24 ± 0.80	0.60 ± 0.72	**0.004**
Δ zlen	0.18 ± 0.36	−0.04 ± 0.23	**<0.0001**
Δ zwei	0.31 ± 0.44	0.34 ± 0.38	0.57
Δ zbmi	0.25 ± 0.66	0.65 ± 0.60	**0.003**
Δ zwfl	0.33 ± 0.66	0.58 ± 0.58	**0.015**

**Table 3:** Taxa that are significantly correlated with differences between baseline and endline (light grey), postbiotic group (unshaded), or probiotic group (dark grey) using linear mixed modelling with fixed effects.

Taxon	Term	Estimate	Std. Error	Statistic	*p*. Value	*p*. Adjusted
unchar. taxon in Lachnospiraceae	baseline–endline	−0.0037	0.0012	−3.1932	0.0017	0.0222
*Enterobacter*	baseline–endline	−0.0066	0.0023	−2.8849	0.0042	0.0414
*Haemophilus*	baseline–endline	−0.0020	0.0005	−3.7275	0.0002	0.0053
*Olsenella*	baseline–endline	−0.0003	0.0001	−2.9117	0.0041	0.0414
*Faecalibacterium*	baseline–endline	0.0850	0.0177	4.7982	2.53 × 10^−6^	0.0002
*Escherichia/Shigella*	baseline–endline	−0.0492	0.0084	−5.8807	1.09 × 10^−8^	1.51 × 10^−6^
*Butyricicoccus*	baseline–endline	−0.0020	0.0006	−3.5221	0.0005	0.0098
*Christensenellaceae R-7 group*	baseline–endline	−0.0036	0.0011	−3.1749	0.0017	0.0222
*Bifidobacterium*	baseline–endline	−0.0216	0.0043	−5.0126	1.50 × 10^−6^	0.0001
*Oscillospiraceae UCG-003*	baseline–endline	−0.0007	0.0002	−3.0814	0.0025	0.0271
*Senegalimassilia*	baseline–endline	−0.0008	0.0002	−3.8557	0.0002	0.0052
*Fusicatenibacter*	baseline–endline	−0.0011	0.0004	−2.9348	0.0039	0.0410
*Muribaculaceae*	baseline–endline	−0.0015	0.0005	−2.8291	0.0050	0.0474
*Alistipes*	baseline–endline	−0.0061	0.0015	−4.1678	5.19 × 10^−5^	0.0021
*Catenibacterium*	baseline–endline	−0.0028	0.0009	−3.1177	0.0022	0.0252
*Collinsella*	baseline–endline	−0.0038	0.0005	−6.9461	1.09 × 10^−10^	3.00 × 10^−8^
*Turicibacter*	baseline–endline	−0.0013	0.0004	−3.1566	0.0019	0.0232
*Barnesiella*	baseline–endline	−0.0012	0.0003	−3.6632	0.0003	0.0073
*Monoglobus*	baseline–endline	−0.0022	0.0007	−3.2575	0.0014	0.0222
*Eubacterium hallii group*	baseline–endline	−0.0057	0.0015	−3.7308	0.0002	0.0053
*Ruminococcus torques group*	baseline–endline	−0.0044	0.0009	−4.6511	4.97 × 10^−6^	0.0003
*Erysipelotrichaceae UCG-003*	group postbiotic	0.0025	0.0007	3.7791	0.0002	0.0053
unchar. taxon in Lachnospiraceae	group probiotic	0.0064	0.0018	3.4943	0.0006	0.0115
*Faecalibacterium*	group probiotic	−0.0696	0.0220	−3.1552	0.0018	0.0222
*Butyricicoccus*	group probiotic	0.0023	0.0007	3.1835	0.0016	0.0222
*Fusicatenibacter*	group probiotic	0.0018	0.0006	3.2622	0.0014	0.0222
*Eubacterium hallii group*	group probiotic	0.0073	0.0019	3.8433	0.0001	0.0051
*Erysipelotrichaceae UCG-003*	group probiotic	0.0028	0.0007	4.1647	5.26 × 10^−5^	0.0021
*Ruminococcus torques group*	group probiotic	0.0037	0.0012	3.1970	0.0015	0.0222

## Data Availability

The datasets analyzed during the current study are available from the corresponding author on reasonable request. Sequence data has been deposited in the Sequence Read Archive under Bioproject number PRJNA1065733 (https://www.ncbi.nlm.nih.gov/bioproject/PRJNA1065733/, accessed on 18 June 2025).

## Supplementary Materials

The following supporting information can be downloaded at https://www.mdpi.com/article/10.3390/foods14132218/s1, Figure S1. Paired boxplots of anthropometric measures before and after the 6-month intervention; Figure S2. Boxplots of the three taxa that were different between stunted and non-stunted children at baseline. Modified from [10]; Figure S3. Paired boxplots of taxa that were significant after linear mixed modelling (Table 3); only those taxa are plotted here that are not yet in Figure 3 or Figure 4. (A) *Alistipes*, (B) *Barnesiella*, (C) *Butyricicoccus*, (D) *Christensenellaceae R-7 group*, (E) *Erysipelotrichaceae UCG-003*, (F) *Eubacterium hallii group*, (G) *Monoglobus*, (H) *Olsenella*, (I) *Oscillospiraceae UCG-003*, (J) *Senegalimassilia*, (K) *Turicibacter*; Table S1. ANOVA results of microbiota changes in the three different treatment groups; Table S2. ANOVA results of microbiota changes in the three different treatment groups segregated by stunted and non-stunted children; Table S3. *Q*- and rho-values for the Spearman correlations of Figure 5. Only significant values are provided.

## Abbreviations

The following abbreviations are used in this manuscript:

ASVs	Amplified sequence variants
CFUs	Colony-forming units
ENT	East Nusa Tenggara
FDR	False discovery rate
SCFAs	Short-chain fatty acids
WHO	World Health Organization
zbmi	BMI for age
zlen	Height-for-age Z-score
zwei	Weight for age
zwfl	Weight for length
