# Supplementary material for: Effect of a 6-Month Functional Food Intervention on the Microbiota of Stunted Children in East Nusa Tenggara, Indonesia—A Randomized Placebo-Controlled Parallel Trial
Source: Foods. 2025 Jun 24;14(13):2218. doi: 10.3390/foods14132218 (PMC12248618; doi:10.3390/foods14132218)
Supplement: Supplementary file 1 [file foods-14-02218-s001.zip › Suppl Table 1.pdf]

ANOVA

Supplemental Table 1a

DESCRIPTION — WEIGHT

Group	Count	Sum	Mean	Variance
placebo	47	54.67	1.163191	0.2182
postbiotic	55	73.6	1.338182	0.697589
probiotic	55	76.42	1.389455	0.588727

ANOVA

Sources	SS	df	MS	F	P value	Eta-sq
Between Groups	1.39776	2	0.69888	1.353834	0.261304	3.054771
Within Groups	79.49832	154	0.516223			
Total	80.89608	156				

Supplemental Table 1b

DESCRIPTION — HEIGHT

Group	Count	Sum	Mean	Variance
placebo	47	159.2	3.387234	1.632877
postbiotic	55	175.5	3.190909	2.380842
probiotic	55	184.4	3.352727	0.964761

ANOVA

Sources	SS	df	MS	F	P value	Eta-sq
Between Groups	1.158745	2	0.579372	0.348835	0.706065	3.054771
Within Groups	255.7749	154	1.660876			
Total	256.9336	156				

Supplemental Table 1c

DESCRIPTION — BMI

Group	Count	Sum	Mean	Variance
placebo	47	12.1	0.257447	0.377106
postbiotic	55	26.22	0.476727	0.671759
probiotic	55	26.71	0.485636	0.750992

ANOVA

Sources	SS	df	MS	F	P value	Eta-sq
Between Groups	1.650568	2	0.825284	1.349542	0.262409	3.054771
Within Groups	94.17546	154	0.611529			
Total	95.82603	156				

Supplemental Table 1d

DESCRIPTION — zlen

Group	Count	Sum	Mean	Variance
placebo	47	6.12	0.130213	0.098033
postbiotic	55	0.58	0.010545	0.137942
probiotic	55	4.11	0.074727	0.06804

ANOVA

Sources	SS	df	MS	F	P value	Eta-sq
Between Groups	0.365841	2	0.182921	1.801994	0.16843	3.054771
Within Groups	15.63255	154	0.10151			
Total	15.99839	156				

Supplemental Table 1e

DESCRIPTION — zwei

Group	Count	Sum	Mean	Variance
placebo	47	13.33	0.283617	0.092971
postbiotic	55	17.68	0.321455	0.236502
probiotic	55	19.85	0.360909	0.164301

ANOVA

Sources	SS	df	MS	F	P value	Eta-sq
Between Groups	0.151928	2	0.075964	0.45133	0.63762	3.054771
Within Groups	25.92002	154	0.168312			
Total	26.07195	156				

Supplemental Table 1f

DESCRIPTION — zbmi

Group	Count	Sum	Mean	Variance
placebo	47	12.98	0.27617	0.274663
postbiotic	55	26.67	0.484909	0.520166
probiotic	55	24.43	0.444182	0.457188

ANOVA

Sources	SS	df	MS	F	P value	Eta-sq
Between Groups	1.214141	2	0.60707	1.429239	0.24265	3.054771
Within Groups	65.41162	154	0.424751			
Total	66.62576	156				

Supplemental Table 1g

DESCRIPTION — zwfl

Group	Count	Sum	Mean	Variance
placebo	47	16.18	0.344255	0.253677
postbiotic	55	28.36	0.515636	0.490888
probiotic	55	27.1	0.492727	0.442435

ANOVA

Sources	SS	df	MS	F	P value	Eta-sq
Between Groups	0.856665	2	0.428332	1.062747	0.348026	3.054771
Within Groups	62.06859	154	0.403043			
Total	62.92526	156				
