# Supplementary material for: Effect of a 6-Month Functional Food Intervention on the Microbiota of Stunted Children in East Nusa Tenggara, Indonesia—A Randomized Placebo-Controlled Parallel Trial
Source: Foods. 2025 Jun 24;14(13):2218. doi: 10.3390/foods14132218 (PMC12248618; doi:10.3390/foods14132218)
Supplement: Supplementary file 1 [file foods-14-02218-s001.zip › Suppl Table 2.pdf]

ANOVA Supplemental Table 2a

WEIGHT

DESCRIPTION alpha 0.05

Group	Count	Sum	Mean	Variance	SS	Std Err	Lower	Upper
plac_stunted	28.00	33.07	1.18	0.24	6.43	0.13	0.920	1.442
plac_normal	19.00	21.60	1.14	0.20	3.58	0.16	0.820	1.454
post_stunted	25.00	26.00	1.04	0.73	17.44	0.14	0.764	1.316
post_normal	30.00	47.60	1.59	0.56	16.15	0.13	1.335	1.839
pro_stunted	26.00	31.42	1.21	0.42	10.42	0.14	0.938	1.479
pro_normal	29.00	45.00	1.55	0.71	19.75	0.13	1.295	1.808

ANOVA

Sources	SS	df	MS	F	P value	Eta-sq	RMSSE	Omega Sq
Between Groups	7.11	5.00	1.42	2.91	0.02	0.09	0.3267	0.0573
Within Groups	73.79	151.00	0.49					
Total	80.90	156.00	0.52					

WEIGHT

TUKEY HSD/KRAMER alpha 0.05

group	mean	n	ss	df	q-crit
plac_stunted	1.18	28	6.4309		
plac_normal	1.14	19	3.5842		
post_stunted	1.04	25	17.4400		
post_normal	1.59	30	16.1547		
pro_stunted	1.21	26	10.4235		
pro_normal	1.55	29	19.7524		
		157	73.7857	151	4.0825

Q TEST

group 1	group 2	mean	std err	q-stat	lower	upper	p-value	mean-crit	Cohen d
plac_stunted	plac_normal	0.044	0.147	0.301	-0.556	0.644	1.000	0.600	0.063
plac_stunted	post_stunted	0.141	0.136	1.037	-0.414	0.696	0.977	0.555	0.202
plac_stunted	post_normal	0.406	0.130	3.123	-0.125	0.936	0.240	0.530	0.580
plac_stunted	pro_stunted	0.027	0.135	0.203	-0.522	0.577	1.000	0.550	0.039
plac_stunted	pro_normal	0.371	0.131	2.830	-0.164	0.905	0.346	0.535	0.530
plac_normal	post_stunted	0.097	0.150	0.644	-0.517	0.711	0.998	0.614	0.139
plac_normal	post_normal	0.450	0.145	3.104	-0.142	1.041	0.246	0.592	0.643
plac_normal	pro_stunted	0.072	0.149	0.480	-0.537	0.681	0.999	0.609	0.102
plac_normal	pro_normal	0.415	0.146	2.844	-0.181	1.010	0.341	0.596	0.594
post_stunted	post_normal	0.547	0.134	4.084	0.000	1.093	0.050	0.546	0.782
post_stunted	pro_stunted	0.168	0.138	1.217	-0.397	0.734	0.955	0.565	0.241
post_stunted	pro_normal	0.512	0.135	3.793	-0.039	1.062	0.085	0.551	0.732
post_normal	pro_stunted	0.378	0.132	2.856	-0.162	0.919	0.336	0.541	0.541
post_normal	pro_normal	0.035	0.129	0.271	-0.491	0.560	1.000	0.525	0.050
pro_stunted	pro_normal	0.343	0.133	2.571	-0.202	0.888	0.457	0.545	0.491

Supplemental Table 2b

HEIGHT

DESCRIPTION alpha 0.05

Group	Count	Sum	Mean	Variance	SS	Std Err	Lower	Upper
plac_stunted	28.00	98.40	3.51	2.03	54.89	0.24	3.037	3.992
plac_normal	19.00	60.80	3.20	1.06	19.10	0.29	2.621	3.779
post_stunted	25.00	87.50	3.50	4.16	99.82	0.26	2.995	4.005
post_normal	30.00	88.00	2.93	0.84	24.37	0.23	2.472	3.394
pro_stunted	26.00	94.20	3.62	1.04	26.09	0.25	3.128	4.118
pro_normal	29.00	90.20	3.11	0.80	22.41	0.24	2.641	3.579

ANOVA

Sources	SS	df	MS	F	P value	Eta-sq	RMSSE	Omega Sq
Between Groups	10.26	5.00	2.05	1.26	0.29	0.04	0.2127	0.0081
Within Groups	246.67	151.00	1.63					
Total	256.93	156.00	1.65					

HEIGHT

TUKEY HSD/KRAMER alpha 0.05

group	mean	n	ss	df	q-crit
plac_stunted	3.51	28	54.894		
plac_normal	3.20	19	19.100		
post_stunted	3.50	25	99.820		
post_normal	2.93	30	24.367		
pro_stunted	3.62	26	26.086		
pro_normal	3.11	29	22.407		
		157	246.674	151	4.0825

Q TEST

group 1	group 2	mean	std err	q-stat	lower	upper	p-value	mean-crit	Cohen d
plac_stunted	plac_normal	0.314	0.269	1.170	-0.782	1.411	0.962	1.097	0.246
plac_stunted	post_stunted	0.014	0.249	0.057	-1.001	1.030	1.000	1.015	0.011
plac_stunted	post_normal	0.581	0.237	2.446	-0.389	1.550	0.514	0.970	0.455
plac_stunted	pro_stunted	0.109	0.246	0.442	-0.896	1.114	1.000	1.005	0.085
plac_stunted	pro_normal	0.404	0.239	1.687	-0.574	1.381	0.840	0.978	0.316
plac_normal	post_stunted	0.300	0.275	1.091	-0.823	1.423	0.972	1.123	0.235
plac_normal	post_normal	0.267	0.265	1.006	-0.815	1.348	0.980	1.082	0.209
plac_normal	pro_stunted	0.423	0.273	1.551	-0.691	1.537	0.882	1.114	0.331
plac_normal	pro_normal	0.090	0.267	0.336	-0.999	1.179	1.000	1.089	0.070
post_stunted	post_normal	0.567	0.245	2.315	-0.432	1.566	0.575	0.999	0.443
post_stunted	pro_stunted	0.123	0.253	0.486	-0.910	1.157	0.999	1.033	0.096
post_stunted	pro_normal	0.390	0.247	1.580	-0.617	1.397	0.874	1.007	0.305
post_normal	pro_stunted	0.690	0.242	2.848	-0.299	1.678	0.339	0.989	0.540
post_normal	pro_normal	0.177	0.235	0.752	-0.784	1.138	0.995	0.961	0.138
pro_stunted	pro_normal	0.513	0.244	2.101	-0.484	1.509	0.674	0.996	0.401

Supplemental Table 2c

BMI

DESCRIPTION alpha 0.05

Group	Count	Sum	Mean	Variance	SS	Std Err	Lower	Upper
plac_stunted	28.00	8.28	0.30	0.43	11.74	0.14	0.014	0.578
plac_normal	19.00	3.82	0.20	0.31	5.51	0.17	-0.142	0.544
post_stunted	25.00	3.11	0.12	0.67	16.17	0.15	-0.174	0.423
post_normal	30.00	23.11	0.77	0.50	14.42	0.14	0.498	1.043
pro_stunted	26.00	7.21	0.28	0.87	21.76	0.15	-0.016	0.570
pro_normal	29.00	19.50	0.67	0.59	16.65	0.14	0.395	0.950

ANOVA

Sources	SS	df	MS	F	P value	Eta-sq	RMSSE	Omega Sq
Between Groups	9.58	5.00	1.92	3.36	0.01	0.10	0.3512	0.0698
Within Groups	86.24	151.00	0.57					
Total	95.83	156.00	0.61					

BMI

TUKEY HSD/KRAMER alpha 0.05

group	mean	n	ss	df	q-crit
plac_stunted	0.30	28	11.737		
plac_normal	0.20	19	5.509		
post_stunted	0.12	25	16.166		
post_normal	0.77	30	14.419		
pro_stunted	0.28	26	21.761		
pro_normal	0.67	29	16.653		
		157	86.244	151	4.0825

Q TEST

group 1	group 2	mean	std err	q-stat	lower	upper	p-value	mean-crit	Cohen d
plac_stunted	plac_normal	0.095	0.159	0.596	-0.554	0.743	0.998	0.648	0.125
plac_stunted	post_stunted	0.171	0.147	1.165	-0.429	0.772	0.963	0.600	0.227
plac_stunted	post_normal	0.475	0.140	3.380	-0.099	1.048	0.166	0.573	0.628
plac_stunted	pro_stunted	0.018	0.146	0.126	-0.576	0.613	1.000	0.594	0.024
plac_stunted	pro_normal	0.377	0.142	2.661	-0.201	0.955	0.418	0.578	0.498
plac_normal	post_stunted	0.077	0.163	0.471	-0.587	0.741	0.999	0.664	0.101
plac_normal	post_normal	0.569	0.157	3.633	-0.070	1.209	0.111	0.640	0.753
plac_normal	pro_stunted	0.076	0.161	0.473	-0.582	0.735	0.999	0.658	0.101
plac_normal	pro_normal	0.471	0.158	2.988	-0.173	1.115	0.286	0.644	0.624
post_stunted	post_normal	0.646	0.145	4.463	0.055	1.237	0.023	0.591	0.855
post_stunted	pro_stunted	0.153	0.150	1.022	-0.458	0.764	0.979	0.611	0.202
post_stunted	pro_normal	0.548	0.146	3.758	-0.047	1.143	0.090	0.595	0.725
post_normal	pro_stunted	0.493	0.143	3.443	-0.092	1.078	0.151	0.585	0.652
post_normal	pro_normal	0.098	0.139	0.704	-0.470	0.666	0.996	0.568	0.130
pro_stunted	pro_normal	0.395	0.144	2.738	-0.194	0.984	0.385	0.589	0.523

Supplemental Table 2d

zlen

DESCRIPTION — alpha 0.05

Group	Count	Sum	Mean	Variance	SS	Std Err	Lower	Upper
plac_stunted	28.00	5.79	0.21	0.11	3.01	0.06	0.093	0.320
plac_normal	19.00	0.33	0.02	0.06	1.09	0.07	-0.120	0.155
post_stunted	25.00	3.49	0.14	0.23	5.62	0.06	0.020	0.260
post_normal	30.00	-2.91	-0.10	0.04	1.06	0.06	-0.207	0.013
pro_stunted	26.00	4.63	0.18	0.06	1.46	0.06	0.060	0.296
pro_normal	29.00	-0.52	-0.02	0.06	1.69	0.06	-0.129	0.094

ANOVA

Sources	SS	df	MS	F	P value	Eta-sq	RMSSE	Omega Sq
Between Groups	2.06	5.00	0.41	4.47	0.00	0.13	0.399	0.099
Within Groups	13.94	151.00	0.09					
Total	16.00	156.00	0.10					

zlen

TUKEY HSD/KRAMER — alpha 0.05

group	mean	n	ss	df	q-crit
plac_stunted	0.21	28	3.0092		
plac_normal	0.02	19	1.0942		
post_stunted	0.14	25	5.6245		
post_normal	-0.10	30	1.0610		
pro_stunted	0.18	26	1.4610		
pro_normal	-0.02	29	1.6865		
		157	13.9364	151	4.0825

Q TEST

group 1	group 2	mean	std err	q-stat	lower	upper	p-value	mean-crit	Cohen d
plac_stunted	plac_normal	0.189	0.064	2.967	-0.071	0.450	0.294	0.261	0.623
plac_stunted	post_stunted	0.067	0.059	1.137	-0.174	0.308	0.966	0.241	0.221
plac_stunted	post_normal	0.304	0.056	5.382	0.073	0.534	0.003	0.230	1.000
plac_stunted	pro_stunted	0.029	0.059	0.491	-0.210	0.268	0.999	0.239	0.094
plac_stunted	pro_normal	0.225	0.057	3.948	-0.008	0.457	0.064	0.232	0.740
plac_normal	post_stunted	0.122	0.065	1.870	-0.145	0.389	0.772	0.267	0.402
plac_normal	post_normal	0.114	0.063	1.816	-0.143	0.371	0.793	0.257	0.376
plac_normal	pro_stunted	0.161	0.065	2.479	-0.104	0.425	0.499	0.265	0.529
plac_normal	pro_normal	0.035	0.063	0.557	-0.224	0.294	0.999	0.259	0.116
post_stunted	post_normal	0.237	0.058	4.067	-0.001	0.474	0.051	0.237	0.779
post_stunted	pro_stunted	0.038	0.060	0.639	-0.207	0.284	0.998	0.246	0.127
post_stunted	pro_normal	0.158	0.059	2.687	-0.082	0.397	0.406	0.239	0.519
post_normal	pro_stunted	0.275	0.058	4.779	0.040	0.510	0.012	0.235	0.905
post_normal	pro_normal	0.079	0.056	1.413	-0.149	0.307	0.917	0.228	0.260
pro_stunted	pro_normal	0.196	0.058	3.378	-0.041	0.433	0.167	0.237	0.645

Supplemental Table 2e

zwei

DESCRIPTION — Alpha 0.05

Group	Count	Sum	Mean	Variance	SS	Std Err	Lower	Upper
plac_stunted	28.00	10.04	0.36	0.10	2.74	0.08	0.207	0.510
plac_normal	19.00	3.29	0.17	0.06	1.15	0.09	-0.011	0.357
post_stunted	25.00	5.26	0.21	0.30	7.16	0.08	0.050	0.371
post_normal	30.00	12.42	0.41	0.17	5.04	0.07	0.267	0.561
pro_stunted	26.00	8.84	0.34	0.19	4.74	0.08	0.183	0.497
pro_normal	29.00	11.01	0.38	0.15	4.11	0.08	0.231	0.529

ANOVA

Sources	SS	df	MS	F	P value	Eta-sq	RMSSE	Omega Sq
Between Groups	1.13	5.00	0.23	1.37	0.24	0.04	0.240	0.012
Within Groups	24.94	151.00	0.17					
Total	26.07	156.00	0.17					

zwei

TUKEY HSD/KRAMER — alpha 0.05

group	mean	n	ss	df	q-crit
plac_stunted	0.36	28	2.7387		
plac_normal	0.17	19	1.1488		
post_stunted	0.21	25	7.1623		
post_normal	0.41	30	5.0435		
pro_stunted	0.34	26	4.7440		
pro_normal	0.38	29	4.1067		
		157	24.9441	151	4.0825

Q TEST

group 1	group 2	mean	std err	q-stat	lower	upper	p-value	mean-crit	Cohen d
plac_stunted	plac_normal	0.185	0.085	2.171	-0.163	0.534	0.642	0.349	0.456
plac_stunted	post_stunted	0.148	0.079	1.874	-0.175	0.471	0.771	0.323	0.365
plac_stunted	post_normal	0.055	0.076	0.734	-0.253	0.364	0.995	0.308	0.136
plac_stunted	pro_stunted	0.019	0.078	0.237	-0.301	0.338	1.000	0.320	0.046
plac_stunted	pro_normal	0.021	0.076	0.277	-0.290	0.332	1.000	0.311	0.052
plac_normal	post_stunted	0.037	0.087	0.426	-0.320	0.394	1.000	0.357	0.092
plac_normal	post_normal	0.241	0.084	2.858	-0.103	0.585	0.335	0.344	0.593
plac_normal	pro_stunted	0.167	0.087	1.923	-0.187	0.521	0.751	0.354	0.410
plac_normal	pro_normal	0.206	0.085	2.434	-0.140	0.553	0.520	0.346	0.508
post_stunted	post_normal	0.204	0.078	2.616	-0.114	0.521	0.437	0.318	0.501
post_stunted	pro_stunted	0.130	0.081	1.610	-0.199	0.458	0.865	0.329	0.319
post_stunted	pro_normal	0.169	0.078	2.158	-0.151	0.489	0.648	0.320	0.416
post_normal	pro_stunted	0.074	0.077	0.961	-0.240	0.388	0.984	0.314	0.182
post_normal	pro_normal	0.034	0.075	0.459	-0.271	0.340	1.000	0.306	0.085
pro_stunted	pro_normal	0.040	0.078	0.511	-0.277	0.357	0.999	0.317	0.098

Supplemental Table 2f

zbmi

DESCRIPTION — Alpha 0.05

Group	Count	Sum	Mean	Variance	SS	Std Err	Lower	Upper
plac_stunted	28.00	8.31	0.30	0.32	8.64	0.12	0.062	0.532
plac_normal	19.00	4.67	0.25	0.22	3.96	0.14	-0.039	0.531
post_stunted	25.00	4.45	0.18	0.50	12.01	0.13	-0.071	0.427
post_normal	30.00	22.22	0.74	0.41	11.77	0.11	0.514	0.968
pro_stunted	26.00	7.36	0.28	0.54	13.49	0.12	0.039	0.527
pro_normal	29.00	17.07	0.59	0.35	9.92	0.12	0.358	0.819

ANOVA

Sources	SS	df	MS	F	P value	Eta-sq	RMSSE	Omega Sq
Between Groups	6.84	5.00	1.37	3.46	0.01	0.10	0.354	0.073
Within Groups	59.79	151.00	0.40					
Total	66.63	156.00	0.43					

zbmi

TUKEY HSD/KRAMER — alpha 0.05

group	mean	n	ss	df	q-crit
plac_stunted	0.296785714	28	8.6438		
plac_normal	0.245789474	19	3.9613		
post_stunted	0.178	25	12.0052		
post_normal	0.740666667	30	11.7666		
pro_stunted	0.283076923	26	13.4872		
pro_normal	0.58862069	29	9.9211		
		157	59.7852	151	4.0825

Q TEST

group 1	group 2	mean	std err	q-stat	lower	upper	p-value	mean-crit	Cohen d
plac_stunted	plac_normal	0.051	0.132	0.386	-0.489	0.591	1.000	0.540	0.081
plac_stunted	post_stunted	0.119	0.122	0.970	-0.381	0.619	0.983	0.500	0.189
plac_stunted	post_normal	0.444	0.117	3.797	-0.033	0.921	0.084	0.477	0.705
plac_stunted	pro_stunted	0.014	0.121	0.113	-0.481	0.508	1.000	0.495	0.022
plac_stunted	pro_normal	0.292	0.118	2.476	-0.189	0.773	0.501	0.481	0.464
plac_normal	post_stunted	0.068	0.135	0.501	-0.485	0.621	0.999	0.553	0.108
plac_normal	post_normal	0.495	0.130	3.794	-0.038	1.027	0.085	0.533	0.786
plac_normal	pro_stunted	0.037	0.134	0.278	-0.511	0.586	1.000	0.548	0.059
plac_normal	pro_normal	0.343	0.131	2.611	-0.193	0.879	0.440	0.536	0.545
post_stunted	post_normal	0.563	0.120	4.670	0.071	1.055	0.015	0.492	0.894
post_stunted	pro_stunted	0.105	0.125	0.843	-0.404	0.614	0.991	0.509	0.167
post_stunted	pro_normal	0.411	0.121	3.382	-0.085	0.906	0.166	0.496	0.653
post_normal	pro_stunted	0.458	0.119	3.838	-0.029	0.944	0.078	0.487	0.727
post_normal	pro_normal	0.152	0.116	1.312	-0.321	0.625	0.939	0.473	0.242
pro_stunted	pro_normal	0.306	0.120	2.543	-0.185	0.796	0.470	0.491	0.486

Supplemental Table 2g

zwfl

DESCRIPTION — Alpha 0.05

Group	Count	Sum	Mean	Variance	SS	Std Err	Lower	Upper
plac_stunted	29.00	11.25	0.39	0.30	8.31	0.11	0.162	0.614
plac_normal	19.00	5.27	0.28	0.18	3.22	0.14	-0.002	0.557
post_stunted	25.00	6.22	0.25	0.50	12.11	0.12	0.005	0.492
post_normal	30.00	22.14	0.74	0.38	11.13	0.11	0.516	0.960
pro_stunted	26.00	9.28	0.36	0.54	13.45	0.12	0.118	0.596
pro_normal	29.00	17.82	0.61	0.34	9.53	0.11	0.388	0.841

ANOVA

Sources	SS	df	MS	F	P value	Eta-sq	RMSSE	Omega Sq
Between Groups	5.18	5.00	1.04	2.73	0.02	0.08	0.318	0.052
Within Groups	57.76	152.00	0.38					
Total	62.94	157.00	0.40					

zwfl

TUKEY HSD/KRAMER — alpha 0.05

group	mean	n	ss	df	q-crit
plac_stunted	0.387931034	29	8.3103		
plac_normal	0.277368421	19	3.2186		
post_stunted	0.2488	25	12.1113		
post_normal	0.738	30	11.1333		
pro_stunted	0.356923077	26	13.4526		
pro_normal	0.614482759	29	9.5295		
		158	57.7555	152	4.0821

Q TEST

group 1	group 2	mean	std err	q-stat	lower	upper	p-value	mean-crit	Cohen d
plac_stunted	plac_normal	0.111	0.129	0.859	-0.415	0.636	0.990	0.525	0.179
plac_stunted	post_stunted	0.139	0.119	1.170	-0.346	0.625	0.962	0.486	0.226
plac_stunted	post_normal	0.350	0.114	3.084	-0.113	0.813	0.253	0.463	0.568
plac_stunted	pro_stunted	0.031	0.118	0.263	-0.450	0.512	1.000	0.481	0.050
plac_stunted	pro_normal	0.227	0.114	1.979	-0.241	0.694	0.727	0.467	0.368
plac_normal	post_stunted	0.029	0.133	0.215	-0.513	0.570	1.000	0.542	0.046
plac_normal	post_normal	0.461	0.128	3.604	-0.061	0.982	0.117	0.522	0.747
plac_normal	pro_stunted	0.080	0.132	0.605	-0.457	0.617	0.998	0.537	0.129
plac_normal	pro_normal	0.337	0.129	2.620	-0.188	0.862	0.435	0.525	0.547
post_stunted	post_normal	0.489	0.118	4.145	0.007	0.971	0.044	0.482	0.794
post_stunted	pro_stunted	0.108	0.122	0.886	-0.390	0.607	0.989	0.498	0.175
post_stunted	pro_normal	0.366	0.119	3.074	-0.120	0.851	0.256	0.486	0.593
post_normal	pro_stunted	0.381	0.117	3.263	-0.096	0.858	0.198	0.477	0.618
post_normal	pro_normal	0.124	0.114	1.088	-0.340	0.587	0.972	0.463	0.200
pro_stunted	pro_normal	0.258	0.118	2.188	-0.223	0.738	0.634	0.481	0.418
