# Supplementary material for: Effect of a 6-Month Functional Food Intervention on the Microbiota of Stunted Children in East Nusa Tenggara, Indonesia—A Randomized Placebo-Controlled Parallel Trial
Source: Foods. 2025 Jun 24;14(13):2218. doi: 10.3390/foods14132218 (PMC12248618; doi:10.3390/foods14132218)
Supplement: Supplementary file 1 [file foods-14-02218-s001.zip › Suppl Table 3.pdf]

Supplemental Table 3

q -value	uncharacterized taxon of Oscillospiraceae	UCG 001 of family XIII of Anaerovoracaceae	Butyricicoccus	taxon RF39 of order Bacilli	uncharacterized taxon of Prevotellaceae	Oscillospiraceae UCG-003	Rikenellaceae RC9 gut group	Lachnospiraceae UCG-010	Muribaculaceae	Sarcina
change in weight		0.015	0.455	0.846	0.455	0.719	0.073	0.214	0.719	0.880
change in length			0.096	0.096	0.096	0.153	0.373	0.756	0.096	0.096
change in BMI	0.011	0.005	0.024	0.216	0.006	0.090	0.006	0.090	0.090	0.240
change in length-for-age	0.065		0.048	0.048	0.048	0.048	0.213	0.574	0.048	0.081
change in weight-for-age		0.045	0.686	0.893	0.688	0.795	0.172	0.644	0.795	0.841
change in BMI-for-age	0.014	0.004	0.015	0.266	0.014	0.110	0.004	0.116	0.110	0.270
change in weight-for-length	0.022	0.005	0.022	0.302	0.022	0.119	0.010	0.119	0.119	0.302

rho-value	uncharacterized taxon of Oscillospiraceae	UCG 001 of family XIII of Anaerovoracaceae	Butyricicoccus	taxon RF39 of order Bacilli	uncharacterized taxon of Prevotellaceae	Oscillospiraceae UCG-003	Rikenellaceae RC9 gut group	Lachnospiraceae UCG-010	Muribaculaceae	Sarcina
change in weight		0.304					0.256			
change in length			-0.238	-0.233	-0.256				-0.241	-0.228
change in BMI	0.282	0.324	0.261		0.300		0.307	0.217		
change in length-for-age	-0.233		-0.245	-0.260	-0.270	-0.246			-0.254	-0.224
change in weight-for-age		0.282								
change in BMI-for-age	0.277	0.325	0.271		0.284		0.314			
change in weight-for-length	0.268	0.324	0.266		0.263		0.299			
