# Supplementary figures and images for: Effect of a 6-Month Functional Food Intervention on the Microbiota of Stunted Children in East Nusa Tenggara, Indonesia—A Randomized Placebo-Controlled Parallel Trial
Source: Foods. 2025 Jun 24;14(13):2218. doi: 10.3390/foods14132218 (PMC12248618; doi:10.3390/foods14132218)

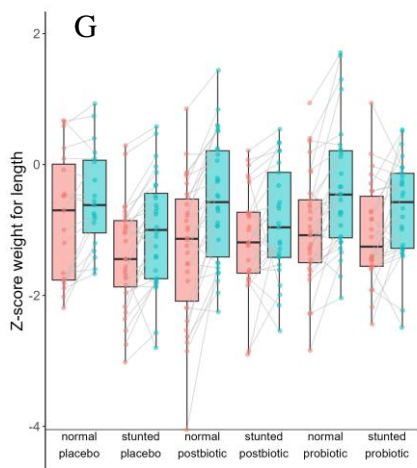

Supplement: Supplementary file 1 [file foods-14-02218-s001.zip › Suppl Fig 1.pdf]

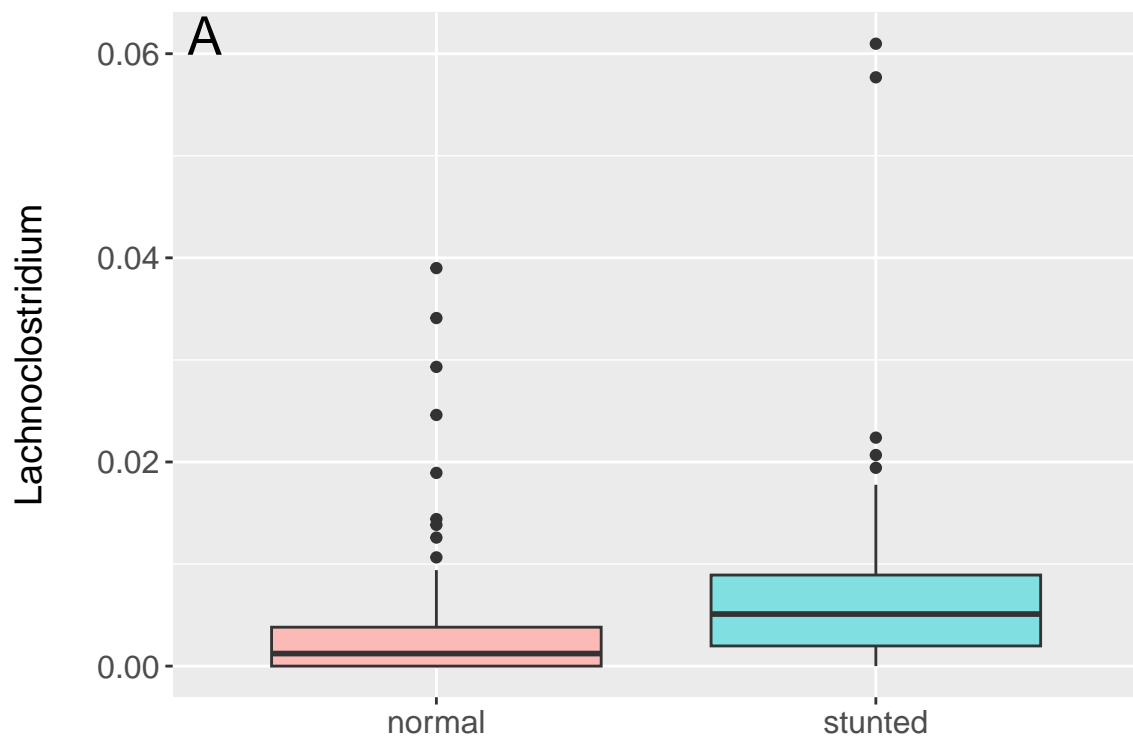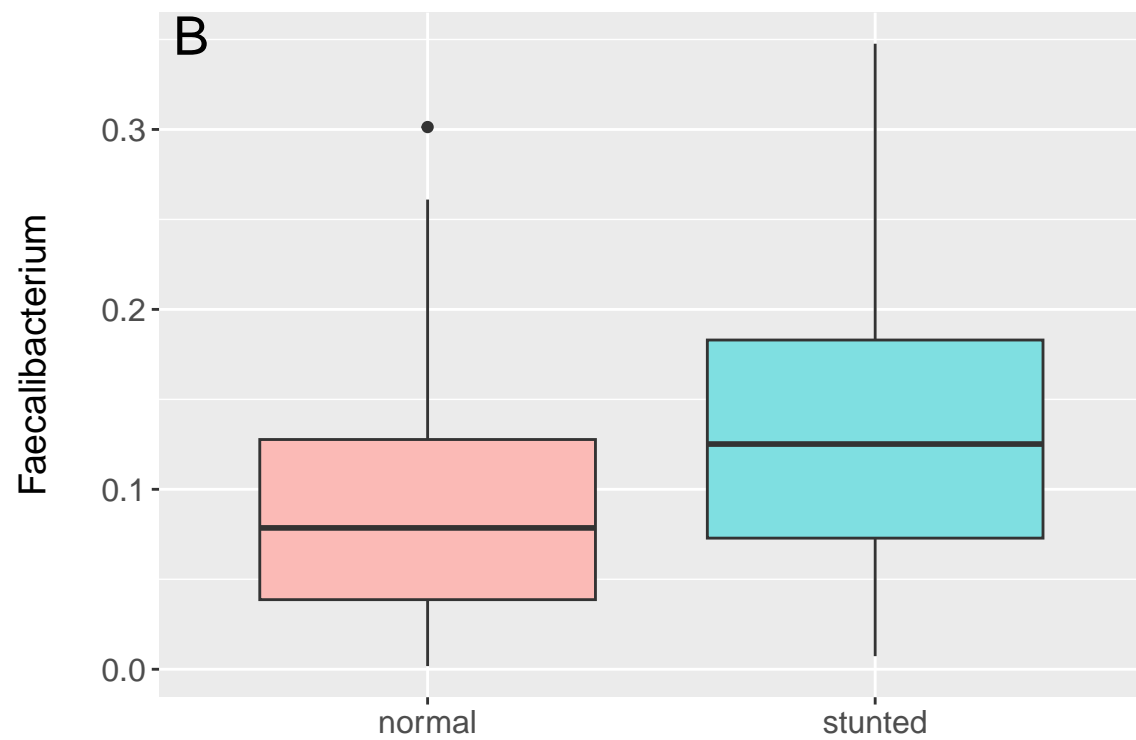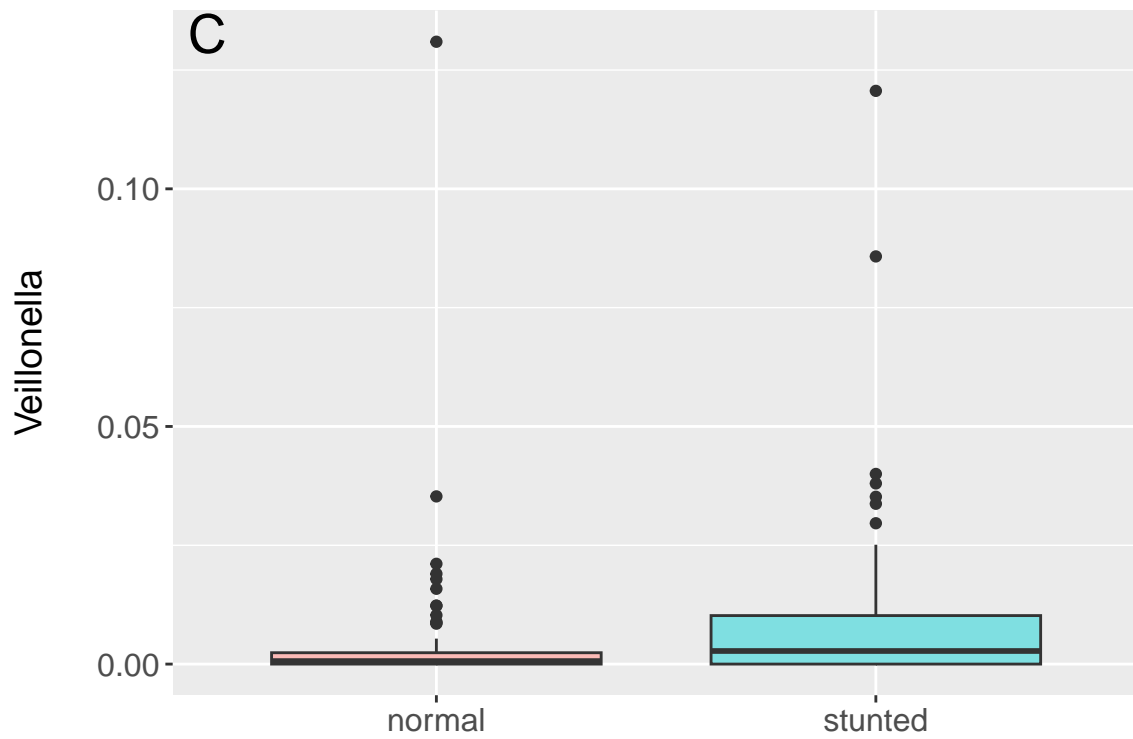

Supplement: Supplementary file 1 [file foods-14-02218-s001.zip › Suppl Fig 2.pdf]

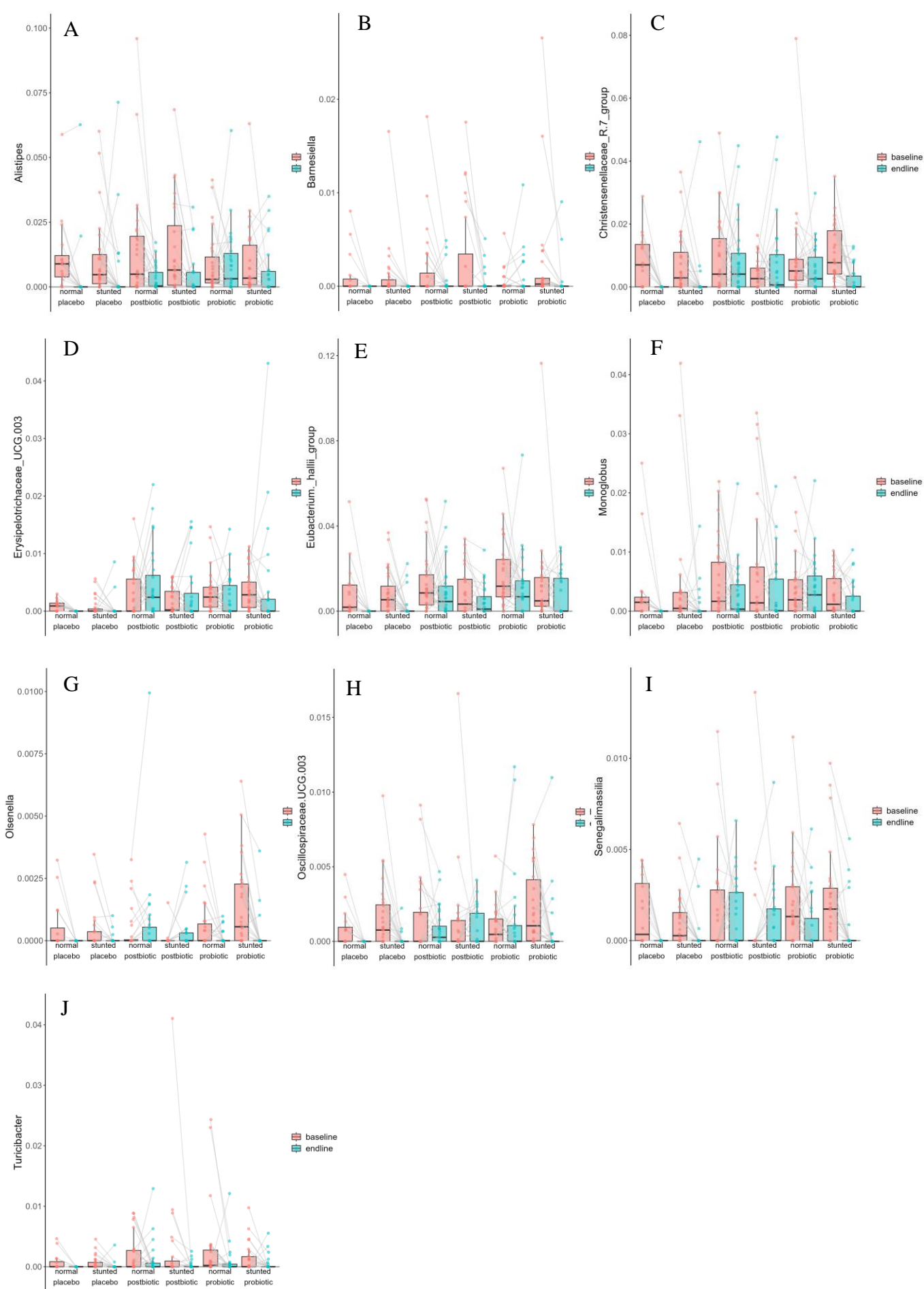

Supplement: Supplementary file 1 [file foods-14-02218-s001.zip › Suppl Fig 3.pdf]
